# What Determines Vaccine Hesitancy: Recommendations from Childhood Vaccine Hesitancy to Address COVID-19 Vaccine Hesitancy

**DOI:** 10.3390/vaccines10010080

**Published:** 2022-01-06

**Authors:** Farren Rodrigues, Suzanne Block, Suruchi Sood

**Affiliations:** Dornsife School of Public Health, Drexel University, Philadelphia, PA 19104, USA; fr88@drexel.edu (F.R.); suzanne.j.block@gmail.com (S.B.)

**Keywords:** vaccine uptake, vaccine hesitancy, COVID-19 vaccination, conceptual model, behavioral and social sciences

## Abstract

Vaccine hesitancy is a prevalent and ongoing issue. However, due to the COVID-19 pandemic, additional attention has been brought to the topic of vaccine hesitancy. Vaccine hesitancy is a threat to the population’s health globally. This article aims to acquire insights from previous literature to determine what works to increase vaccine uptake and how we can apply this knowledge to increase COVID-19 vaccine uptake. Research has focused chiefly on childhood vaccination and the hesitancy of caregivers. After conducting an extensive literature review, we have created a conceptual model of indicators that influence vaccine uptake for health providers and caregivers, which can also be used for vaccine recipients. Overall, the reasons for vaccine hesitancy are complex; therefore, a multifaceted approach is needed to address it. Understanding the factors that affect vaccine hesitancy will aid in addressing hesitancy and, in turn, lead to an increase in vaccine uptake.

## 1. Introduction

The World Health Organization (WHO) listed vaccine hesitancy as one of the top 10 threats to global health in 2019. Vaccine hesitancy is defined as “the reluctance or refusal to vaccinate despite the availability of vaccines [1]”. The reasons for vaccine hesitancy vary and are multifaceted, ranging from behavioral, social, and political factors. Caregivers, vaccine recipients, and frontline health workers (FLWs) must work together to increase the uptake of vaccinations to protect themselves and, in turn, protect their communities. Considering the significance of vaccine hesitancy, this paper aims to acquire insights from previous literature to determine what works to increase vaccine uptake and how we can apply this knowledge in a new conceptual model to increase the uptake of the COVID-19 vaccine. The role of frontline health workers and the importance of interpersonal communication and counseling will also be explored.

If and when the main causal factor to low vaccine uptake is access and availability, domestic and international partners must focus on supply chain issues. However, if the issue is hesitancy regardless of access, one must examine the demand-related factors contributing to low vaccine uptake. The global situation around the COVID-19 vaccine provides a perfect example. In the US, vaccine hesitancy is driven by a lack of demand linked to personal beliefs, stereotypes, and socio-cultural factors [2]. Yet, in much of the Global South, low vaccination rates are fueled by the lack of access to adequate doses of the vaccine to protect entire populations. For example, when COVID-19 vaccines were in development, high-income countries began making deals with pharmaceutical companies for pre-order [3]. Over 80% of the COVID-19 vaccine developed by Pfizer was claimed by the United States, United Kingdom, European Union, and Japan. Even if the vaccine was made available to countries in the Global South (such as Latin America, India, Southeast Asia, and South Africa), there are still costs associated with maintenance, storage, transportation, and distribution [3,4].

### 1.1. Childhood Vaccine Hesitancy

Immunizations are credited with saving the lives of millions of children each year [5]. However, since the introduction of the smallpox vaccine in the 1800s, immunization has also faced controversy and opposition [5,6]. One of the most well-known controversies was the fraudulent and debunked study published in 1998 by Wakefield and colleagues that falsely linked the Measles, Mumps, and Rubella vaccine to autism and helped fuel vaccine hesitancy—and the anti-vaccine movement—among caregivers [7,8]. To this day, although the national coverage of recommended vaccines for children remains steady, a sizeable number of caregivers continue to delay or refuse some or all vaccinations for their children. Consequently, vaccine-preventable disease outbreaks still appear in many parts of the world [7]. In many countries, even when policies, financing, and resources are in place and services are available, a large number of children still fail to complete immunization schedules. In 2020, global vaccination coverage dropped to 83% from 86% in 2019 [9]. It is estimated that 23 million children below one year old were un- or under-vaccinated from basic vaccines [9]. Childhood vaccine hesitancy is a significant barrier towards eliminating vaccine-preventable diseases among young children around the globe. It is clear that as countries shift their focus towards monitoring the COVID-19 pandemic and vaccination programs, that will also impact efforts to address childhood vaccinations.

### 1.2. COVID-19 Vaccine Hesitancy

Currently, the focus of vaccinations has shifted towards COVID-19 vaccination efforts. As of November 2021, the COVID-19 pandemic has seen 248 million cases with over 5 million deaths [10]. In December 2020, based on concerted global efforts, scientists successfully developed vaccines to help control the spread of COVID-19. Unfortunately, vaccine hesitancy has proven to be a major hindrance to COVID-19 vaccination uptake.

A recent systematic review of COVID-19 related to vaccine hesitancy globally found wide variation in vaccine hesitancy between countries, with the overall acceptance rate below 70% [11]. This systematic review also included results from eight separate studies with health care providers and reported vaccine acceptance rates among health care workers ranging from 28% in the Democratic Republic of the Congo to 78% in Israel. The review concludes that addressing the scope of COVID-19 vaccine hesitancy in various countries requires building trust in the vaccination efforts. Additionally, a study by Arce et al. (2021) examined COVID-19 vaccination acceptance among low- and middle-income countries (LMICs) in Latin America, Asia, Africa, and Russia (upper-middle class), and the United States (US) [12]. They surveyed individuals in 10 LMICs (n = 20,176) and compared responses against Russian and US respondents (n = 24,084), identified as two countries leading vaccine research and development. Findings showed that the average COVID-19 vaccine acceptance was higher in LMICs (80% acceptance) compared to Russia (30% acceptance) and the US (65% acceptance). Their findings further revealed various reasons for vaccine hesitancy and most notably included side effect concerns (40.8% in all LMICs, 36.8% in Russia, and 79.3% in the US). Other notable reasons included vaccine effectiveness doubts (29.6% in Russia and 46.8% in the US) and lack of concern related to COVID-19 infection (39.3% in the US). Vaccine hesitancy towards the COVID-19 vaccine is a significant threat to controlling COVID-19 and its various strains globally. Therefore, vaccinations are critical in the road to recovery from the COVID-19 pandemic.

## 2. Methods

We (two of the authors, FR and SB) conducted a literature review of peer-reviewed articles, utilizing Google Scholar and the PubMed database, and grey literature from UNICEF and WHO written in the English language to see approaches that have worked to improve vaccine hesitancy. Overall, the 117 articles were extracted because they addressed vaccine hesitancy and uptake, measurement tools, and quality of service between the health provider and caregiver. The literature review specifically included looking for sets of tools that incorporate direct data collection with providers and caregivers, including the constructs included in the model below. To begin, we conducted a document review and discovered 91 tools that showcase provider and caregiver characteristics. The literature review started with a keyword search for “patient-provider/client-provider interpersonal communication”, which led to a variety of assessment tools in various fields such as counseling and therapy in family planning and drug treatment adherence. Next, when searching for keywords “vaccine hesitancy and interpersonal communication” and “motivation interviewing and vaccine hesitancy”, assessment tools addressing various health concerns and topics were found through Google, Google Scholar, and PubMed. These domains were selected based on available literature on areas where counseling has been examined, such as HIV/AIDS, medication adherence, mental health, reproductive health, and cancer. Inclusion criteria included the presence of valid and reliable tools and questionnaires addressing the quality of service between clients and providers. We presented the conceptual model at capacity-building workshops with professionals from 23 countries in the Europe and Central Asia Region. These colleagues included individuals with affiliations to different health sectors, and all of them had experience in training FLWs. These meetings served as a form of validation for our conceptual model constructs through discussions and feedback based on research and personal experience.

### 2.1. Conceptual Model for Addressing Vaccine Hesitancy

**Figure 1:**
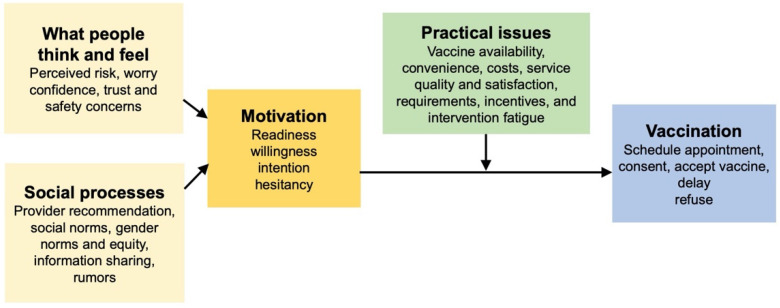
The Increasing Vaccination Model (2017) [13].

**Figure 2:**
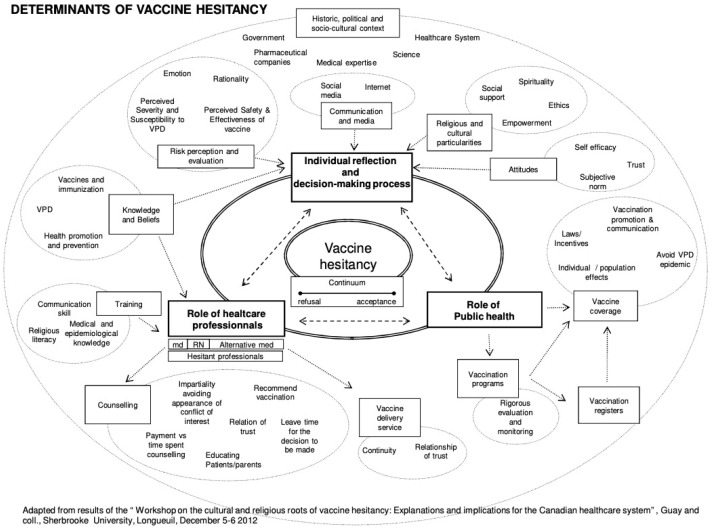
The Vaccine Hesitancy Determinants Model (2011) [7].

**Figure 3:**
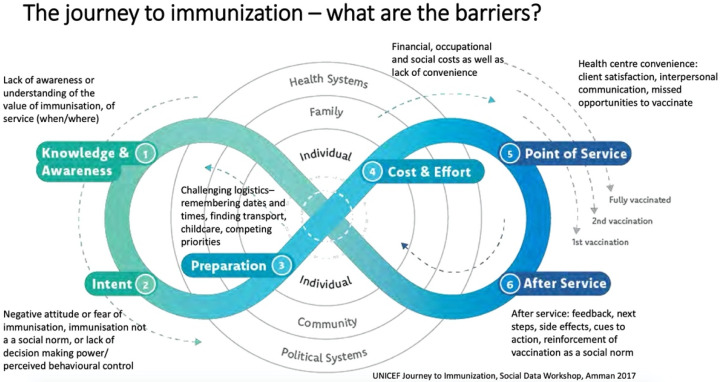
Journey to Immunization–UNICEF (2017) [19].

The creation of our conceptual model below is based on a review of 15 articles specifically relating to vaccine uptake and hesitancy. Our final model combines three existing vaccination models: WHO Increasing Vaccination Model (Figure 1), the SAGE Vaccine Hesitancy Determinants Matrix (Figure 2), and the Journey to Immunization–UNICEF (Figure 3). Each of these models has its strengths and weaknesses. The core strength of each of these models is that they illustrate causal factors associated with vaccine hesitancy.

### 2.2. WHO Increasing Vaccination Model

The 2017 Increasing Vaccination Model showcases the factors that increase vaccination uptake among individuals [13]. This model is set in a context where governments fund some of the cost of vaccinations, or private insurance covers the cost. These factors result from a variety of behaviors by a multitude of different factors [14]. The three main propositions in the model are that vaccination uptake results from what people think and feel and the social processes that lead to motivation and ultimately vaccination uptake (Figure 1).

The first proposition in this model is that an individual’s thoughts and feelings motivate vaccine uptake [14]. This includes perceived risk, hesitancy, confidence, and trust. Social processes come next, motivating the uptake of vaccines and consist of a recommendation from health providers, social and gender norms, information sharing, and rumors. Motivation, as shown in the middle, plays a vital role in predicting vaccine uptake. The practical issues box underscores the barriers to vaccination, but these issues are measurable and can be addressed through vaccination programs. In isolation, these factors might not always have a meaningful impact on vaccine uptake (i.e., individual thoughts and feelings) [14]. Still, their interactions, including the role of practical issues, make this a promising model. Overall, the Increasing Vaccination Model showcases the interrelated factors, resources, and behaviors that affect vaccination uptake.

While very practical, the Increasing Vaccination Model is relatively simple and includes one-way relationships between individual and social processes that can increase vaccination coverage. These one-way relationships fail to consider other interactions that could take place. This model places vaccine hesitancy as part of the “motivation factors” followed by structural issues such as availability and affordability. However, vaccine hesitancy is a complex process that exists despite the availability of vaccines [5,14]. This distinction is important from an intervention perspective. In the model’s current state, it appears that if a provider recommends a vaccine, that will influence motivation, but motivational factors would not impact how a recommendation is perceived. It has been previously demonstrated that an individual who is motivated towards something is selective in how they perceive external factors [15]. In addition, this model does not indicate a relationship between individual thoughts and feelings and social processes. Lastly, as the creators of this model acknowledge, more research is needed to determine the role of social processes on vaccine uptake [14].

While providing ample information on the barriers to vaccination uptake at each level of the socio-ecological model (SEM), the Increasing Vaccination Model falls short of explicating the specific causal linkages between the barriers that cut across the different levels of SEM.

### 2.3. The Vaccine Hesitancy Determinants Model

The Vaccine Hesitancy Determinants Model, created in 2012, was adapted from the results of a discussion held with approximately 40 experts from relevant fields during the “Workshop on the Cultural and Religious Roots of Vaccine Hesitancy: Explanations and implications for the Canadian Healthcare” [7] (Figure 2). This model was based on the 3Cs, which postulates that vaccine hesitancy exists at the intersection of three factors: confidence, complexity, and complacency. The detailed matrix showcases the variety of factors that influence individual behavior regarding vaccine hesitancy [16]. The Vaccine Hesitancy Determinants Model consists of three interrelated areas addressing vaccine hesitancy, including the role of the individual decision-making process, the role of public health, and the role of health professionals. Individual reflection and their decision-making process are influenced by communication and media, religious and cultural characteristics, attitudes, risk perception and evaluation, knowledge, and beliefs. The role of public health influences vaccination programs and coverage. Public health also plays a large role in communicating with the population in an acceptable manner. The role of health professionals is influenced by knowledge, beliefs, and training, and influences counseling and vaccine delivery services [17]. Overall, the model stresses that individual decision-making regarding vaccination is multifaceted and involves emotional, cultural, social, religious, and political factors just as much as it involves cognitive factors.

The Vaccine Hesitancy Determinants Model is a comprehensive approach to understanding multiple causal factors influencing vaccine hesitancy. It has been used widely in research to examine vaccine hesitancy in specific contexts as well as a global phenomenon. While this model has the potential for standardization of the determinants of vaccine hesitancy, there is still a lack of clarity in some of the constructs. For example, previous research has highlighted the challenges of determining vaccine hesitancy at the population level [18]. Additionally, it may be much too complex to understand and apply holistically to intervention research.

### 2.4. The Journey to Immunization–UNICEF

The third model, the Journey to Immunization, created in 2017, follows a caregiver or vaccine recipient’s journey to immunization [19]. This model may also be used to follow the journey of a health care provider since the end goal is immunization (Figure 3). This model spans from the knowledge and awareness of vaccines to what happens after vaccination and allows interventions to focus on the area that needs the most attention. The elements of this model are situated within a socio-ecological framework and include individual, family, community, health care, and political systems. The model applies a steps approach by highlighting six core factors within a vaccination journey. These include knowledge and awareness, intent, preparation, cost and effort, point of service, and after service. Knowledge and awareness address the actual vaccine, disease, and service (such as when, where, and how to get the vaccine). Intent is overcoming the gap between intent to vaccinate and getting vaccinated. Willingness to vaccinate is contributed to attitudes towards the behavior, subjective norms, and perceived behavioral control. Preparation considers the disease, vaccination, and service and determines the logistics of acquiring the vaccination (for example, transportation, cost, and childcare). Cost and effort relate to opportunity, transportation, lost income, uncertainty of service, and social and security costs. Point of service involves all aspects of the vaccination experience (such as client satisfaction and interpersonal communication with health providers). Finally, after service includes some factors such as feedback, next steps, side effects, and reminders.

The Journey to Immunization Model is useful to understand barriers and provides a process-oriented framework for designing future interventions. Still, its cyclical nature makes it difficult to utilize as an evaluation framework.

### 2.5. Front Line Health Workers and Interpersonal Communication and Counselling

UNICEF has found that a key determinant leading to vaccine hesitancy is linked to the quality of communication between health workers and clients. Health workers can be perceived as either trusted sources of information or having insufficient knowledge [20]. Therefore, the final opportunity to be influenced lies in the skills of the FLW. Even though most caregivers support immunizations and immunize their children before entering school, questions and concerns are still customary. Questions and concerns regarding childhood vaccinations and COVID-19 vaccinations can relate to side effects, ingredients, and number of doses [21]. According to WHO, FLWs are seen as influencers and trusted sources of information on vaccines [5]. Therefore, FLWs must be trained and supported to provide accurate information and support to patients. Strengthening health workers’ interpersonal communication and counseling (IPC/C) capacities is one effort to deliver messages and promote vaccine uptake effectively. Interpersonal communication (IPC) specifically focuses on informing and educating caregivers, soon-to-be-caregivers, and vaccine recipients about vaccination. A recommendation from a health provider can move a caregiver or vaccine recipient from hesitant to accepting a vaccine [7,14,16,20,22,23]. Recommendations from a frontline worker may increase confidence, set a social norm, or showcase a direct behavior change method [14].

**Figure 4:**
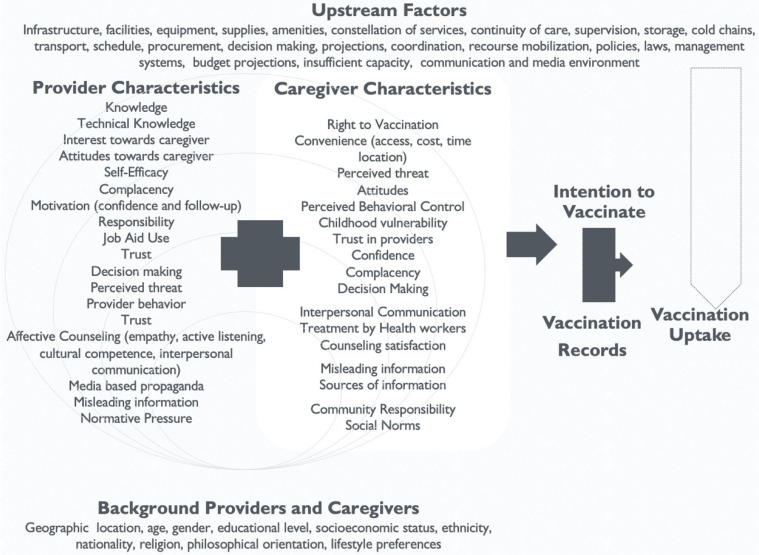
Providers and Caregivers Conceptual Model.

After studying these models and conducting an extensive literature review, the conceptual model below reflects important constructs to consider when addressing vaccine hesitancy (Figure 4). The overarching goal for this model is “To improve demand for and equitable delivery of immunization services with a focus on increased access to and demand for immunization services.” The impact of reducing vaccine hesitancy is measurable through improved vaccination uptake and subsequent declines in the incidence of vaccine-preventable diseases. This is the long-term view of the process. Several proximal factors contribute to the reduction in vaccine hesitancy. Background factors include age, gender, socioeconomic status, and geographic location, to name a few.

Furthermore, other factors can be classified into upstream factors and downstream factors. Upstream factors include facilities, supplies, policies and laws, and communication and media environment. Challenging upstream factors falls within the realm of governments, international organizations, non-governmental organizations (NGOs), and national and local governments. Successful vaccination uptake requires addressing upstream factors that are linked with vaccine uptake but not directly connected with the constructs in our model. The purpose of this model is not to provide a holistic picture but to measure specific intermediate outcomes (indicators), which can then be directly linked to the intention to vaccinate.

The impact of vaccination programs can truly be verified by studying trends in vaccine uptake. This data takes time to collect, is collected at an aggregate level, and does not provide a nuanced look at the ground level. Therefore, one specific key measurable outcome for intervention evaluations can be identified as “intention to vaccinate” and/or “vaccination reported by vaccine recipients”.

This model specifically focuses on provider and caregiver/vaccine recipient factors that influence the intention to vaccinate. This outcome is achievable through a series of related steps. Although most constructs are standalone, some constructs for providers are multidimensional and cannot be directly measured. In these cases, broader topics were identified and subdomains created. For example, the broader topic of affective counseling by providers is measured through empathy, active listening, cultural competence, and interpersonal communication. Combining these questions can help create an affective counseling scale measure, which can then be correlated with other constructs. Additionally, motivation is a function of confidence, follow-up, and interest towards the caregiver.

The constructs under the caregivers’ box in the conceptual model showcase the levels of the SEM. Individual-level constructs include attitudes, perceived threat, social norms, perceived behavioral control, decision-making, complacency, confidence, counseling satisfaction, and interpersonal communication; community-level constructs include trust in providers, caregiver vulnerability, sources of information, misleading information, and community responsibility; finally, at the societal level is convenience and the right to vaccination. This conceptual model is intended to be adaptable to local contexts, and sub-measures that are not relevant to specific contexts can be deleted.

## 3. Results

Vaccine hesitancy is a global issue. Any model seeking to explain causal factors and their relationships must first define the indicators intended to be measured to avoid any misunderstanding. We searched for valid and reliable construct definitions in the conceptual model above, as shown in Table 1 and Table 2 below. The definitions are derived from peer-reviewed articles, academic textbooks, and public health agency websites and reports examining the identified construct.

Overall, it is presumed that addressing the various indicators that impact caregiver behavior will help caregivers decide (intention) to vaccinate their child(ren) or take the vaccine, in the case of vaccine recipients. Positive improvements over time in the intermediate outputs/factors, combined with intent to act on the demand side and improvements in the supply side and upstream factors, will increase vaccination uptake (the impact indicator).

## 4. Discussion

This study aimed to understand what works to address vaccine hesitancy and ultimately increase vaccine uptake. These insights provided the basis for the development of a new conceptual model intended to address this complex issue. After reviewing the literature on the determinants and factors of vaccine hesitancy and vaccine uptake, and the role of FLWs and IPC/counseling, we realized most of the constructs in our conceptual model could be adapted to COVID-19 vaccinations. A primary difference is that instead of only deciding whether to vaccinate their child, individuals also decide for themselves. The reasons for vaccine hesitancy vary in each community or country, and now the public health community is tasked with promoting vaccine uptake among eligible adults, caregivers, and their children aged five years and older [34]. Currently, only 56.5% of the global population is partially vaccinated against COVID-19 [35]. The fewer vaccinated people, the more risk exists to spread disease. Therefore, addressing COVID-19 vaccine hesitancy for those five years old and above is crucial to mitigate the number of coronavirus cases and protect the population’s health.

The core-provider characteristics included in our model can be used to develop role-model provider portfolios for vicarious learning and can be included in training to build the capacity of FLWs, tasked with counseling vaccine recipients. The caregiver or vaccine recipient characteristics can be used to develop tailored interventions for different categories of audiences based on the specific constructs that need to be addressed.

In addition to the three conceptual models presented earlier, previous research has showcased the correlation between sociodemographic factors and vaccine hesitancy [2,36]. Specifically, this includes correlations between vaccine hesitancy and education, income, gender, race, and/or marital status [15,36,37,38,39]. However, some of these factors, including gender, are not consistently correlated with vaccine hesitancy and may be context- or vaccine-specific [36]. To address vaccine hesitancy, there needs to be an understanding of the psychological and social dimensions, with appropriate measures to monitor the shift in behavior accompanied by reliable and valid outcome measures [37,38,39]. The ability to understand these dimensions and contextual adaptation is especially important for COVID-19 vaccine hesitancy.

Although a majority of constructs in our conceptual model can be applied to hesitancy of the COVID-19 vaccine, constructs will need to be adapted to be relevant in certain contexts, while others may not be relevant at all. For instance, when receiving vaccines and waiting in a long line at a grocery store pharmacy, the provider may be unable to engage in extended conversations with vaccine-hesitant caregivers or recipients. As a result, the construct of interpersonal communication and its suggested techniques should be modified. This includes shortening their recommendation or explanation that addresses their concerns. Alternatively, follow-up is a construct that might or might not be applicable for this setting. However, if the vaccine is scheduled and administered in a health clinic with a vaccine recipient’s primary care physician, then these constructs should apply.

Overall, given the setting, organizational needs, or health provider needs, the provider constructs in the newly developed conceptual model will vary in how they interact and their relevance for COVID-19 vaccination. However, regardless of where the vaccine is being administered or how much time the health provider has to administer the vaccine, the provider should always be empathetic, respectful, actively listening to the vaccine recipient, culturally competent, and be able to engage in interpersonal communication. The health provider should also be knowledgeable about the vaccine regarding possible allergies, effectiveness, and potential side effects.

Depending on the setting, the constructs for caregivers or vaccine recipients will also vary in relevance for COVID-19 vaccination. However, special attention should be given to convenience (access, cost, time, and location), trust in providers, and confidence. The vaccine recipient should be able to access the COVID-19 vaccine (either one dose or both) and the subsequent booster dose at a low cost if any. Furthermore, the vaccine recipient needs to have trust in the health provider. Having trust in the health provider can make the vaccination process smoother and provide a sense of ease for the vaccine recipient [40]. Finally, having confidence in the effectiveness and safety of vaccines, the system delivering them, the proficiency of the health providers, and the motivations of the leaders and policymakers is essential in making the vaccination process a seamless one [19].

The conceptual model’s constructs must also be applied to address health provider vaccine hesitancy. Similar to caregivers and vaccine recipients, health provider vaccine hesitancy is not new or isolated to the COVID-19 vaccine and varies by behavioral, social, and other contextual factors. Health providers’ confidence in the vaccines, knowledge, and whether they feel a responsibility to protect and promote the public’s health versus personal autonomy influence their vaccine behaviors and choices [41,42,43,44]. Misleading information or misinformation and low perceived risk (complacency) also contribute to vaccine hesitancy [41,42,43,44]. These constructs must be examined and addressed to promote vaccine uptake among health providers and ensure their role in encouraging their patients to get vaccinated. Specifically, health providers’ knowledge of vaccines [43], confidence in vaccines [41,43,45], and their recommendations are strong predictors of caregiver and vaccine recipients’ decisions regarding vaccination [45].

It is imperative to train health workers and provide them with the skills and techniques to interact with vaccine-hesitant colleagues, caregivers, and vaccine recipients to increase vaccination uptake [5,14]. Organizations trying to tackle the issue of vaccine hesitancy must consider the influx of a variety of factors that affect the demand for immunizations. Health providers should participate in IPC/counseling trainings so that they are prepared to draw upon the conceptual models’ identified constructs to educate their clients, offer strong recommendations, and respond to questions regarding safety, efficacy, and the role in preventing the spread of communicable diseases. Training workshops should be held at a convenient time for them, be culturally appropriate, and in a space that encourages authentic communication [20,27]. Resources should also be provided to health providers to use in the future, including but not limited to job aids, technical assistance, and access to trusted sources for referral (such as the CDC or NIH website). Health providers must encourage the acceptance and uptake of the COVID-19 vaccine to their clients as well as on media platforms and work to negate misleading information [30,46].

Training health workers is a key to addressing vaccine hesitancy among health workers, caregivers, and vaccine recipients, yet there must also be a monitoring and evaluation framework in place to determine the efficacy of this training. Conducting routine monitoring of vaccine hesitancy is critical in identifying concerns regarding vaccinations earlier rather than later [38]. It is crucial since vaccine hesitancy is constantly evolving. It is imperative to better understand the contextual and socio-ecological influences contributing to vaccine hesitancy to engage the population, health providers, and local and national leaders [37,38]. This will lead to an increase in the uptake of the COVID-19 vaccine.

### Strengths, Limitations, and Future Research

This study has several strengths. A key strength is that our conceptual model can be used for situation analysis purposes, to understand and rank the key determinants of vaccine hesitancy among providers and vaccine recipients. Given its adaptability, it can serve a monitoring and evaluation function in various settings. Monitoring looks at the extent to which a program is being implemented according to plan and change is happening. Evaluation measures effectiveness by comparing locations (intervention and comparison) or changes over time. Measuring vaccine hesitancy is imperative to understand if current programs are indeed increasing vaccine uptake.

Another strength is the valid and reliable construct definitions for each potential indicator. Our next step is to develop measurement tools based on our construct definitions. Tools with variables to measure all of the constructs in our model are needed to assess the scope and scale of vaccine hesitancy issues by vaccine type and context. A tool that can be adapted globally would be ideal because it allows comparability across countries [38]; our conceptual model will allow for the necessary modifications.

There are also several limitations to this study. We relied on English language publications to select models and definitions of key terms, given the global nature of COVID 19 related research, it is possible that we failed to consider relevant literature published in other languages. The conceptual model has not yet been formally validated through pilot studies. To date, validation has been based on feedback from specific vaccine experts in the European and Central Asia Region. Moreover, COVID-19 vaccine hesitancy may continue to evolve as new research emerges. As a result, additional constructs that have not been identified in this study should be considered prior to the implementation of this conceptual model. Lastly, we realize that this study does not provide one answer to address vaccine hesitancy and increase vaccine uptake. It is our hope that future research can build on the contents of this paper for future implementation, monitoring, and evaluation purposes.

## 5. Conclusions

The present study integrated findings from previous literature that reported on what works to increase childhood vaccine uptake and how it can be used to promote COVID-19 vaccine uptake. As a result, a new conceptual model was created. Understanding the factors that affect vaccine hesitancy will aid in addressing this issue and, in turn, increase vaccine uptake. Vaccine hesitancy is a complex and constantly changing public health concern. Therefore, a comprehensive model of indicators influencing vaccine hesitancy, in addition to valid and reliable definitions, is crucial to addressing vaccine hesitancy through tailored intervention planning, implementation, and evaluation. This paper is intended to assist with future design, implementation, monitoring, and evaluation. The definitions compiled here can be used for designing new interventions, training health providers, supplementing existing efforts, or for routine monitoring and evaluation purposes. Discrete models tailored to various factors and determinants within local contexts can guide health care workers as well as public health experts in their process. The next step would be to pilot the conceptual model by developing tools for health providers and caregivers.

## Figures and Tables

**Table 1:** Provider construct definitions.

Constructs and Definitions
Active listening The development of a clear understanding of the caregiver’s concern through collaboration and verification of statements as well as clear communication of the provider’s interest in the caregiver’s message through verbal and non-verbal cues [24].
Attitudes (towards caregiver) The perceptions towards caregivers can impact the relationship and whether a desired behavior occurs by the caregiver [20].
Complacency Perceived risks of vaccine-preventable diseases are low, and vaccination is not deemed a necessary preventive action [25].
Confidence Whether the caregiver or health worker trusts the effectiveness and safety of vaccines, the system that delivers them, including the reliability and competence of the health services and health professionals, and the motivations of the policymakers who decide on the needed vaccine [20].
Cultural competence The integration and transformation of knowledge about individuals and groups into specific behaviors are used in cultural settings to increase the quality of services, thereby producing better outcomes [26].
Decision-making The process through which providers and caregivers make pertinent choices based on weighing the pros and cons of a given action [20].
Empathy The ability to understand what others are feeling because you have experienced it yourself or can put yourself in their shoes [20].
Follow-up This includes phone calls, emails, or other means to follow up after a visit or remind caregivers about an upcoming visit. These efforts help promote an increase in uptake and decrease in late vaccination, especially for caregivers who doubt or fear vaccinations [20].
Responsibility An ethical obligation to protect and promote the health-related interests of all child(ren), including the promotion of vaccination [27].
Interest (towards caregivers) Paying attention to and prioritizing caregivers’ and child(ren)’s needs [20].
Interpersonal communication (IPC) The exchange of information; in the case of counseling, this includes the transfer of biomedical or technical information and the complex roles and relationships within which health behaviors are negotiated [20]. Considering IPC in the context of health behaviors means recognizing the interconnections among the roles and relationships of health providers, friends, and family members in their attempts to influence health and illness [28]. This results in clear and effective communication that promotes desired behavioral outcomes.
Job aid use The use of communication material to improve the credibility of information being shared. This is useful since people tend to respond both intellectually and emotionally to external sources of information [20].
Knowledge An individual’s degree of understanding about how to enact a behavior [29].
Media-based propaganda Vaccination-related messages are disseminated by the media to generate compliance and action or inaction among their audience [30].
Misleading information Information that has been manipulated or is inaccurate to promote rumors related to vaccines [31].
Normative pressure Social perceptions regarding the acceptability of a behavior influence whether a behavior is performed [20].
Perceived threat Combined perceived seriousness and perceived susceptibility. Overall, it is a perception that something is dangerous enough for the caregiver to change their behavior [20].
Provider behavior A health care provider’s personal vaccination-related actions and decisions for their own children, family members, and themselves [20].
Respect Facilitating conversation in an open and non-judgmental way to promote partnership between health care providers and caregivers [32].
Self-efficacy An individual’s confidence in their ability to engage in a behavior [29].
Technical knowledge Adequate education, skills, and knowledge of vaccines [20].
Trust The health provider is respectful of the caregiver’s opinions, is clear and understandable, cares about them and wants what is best for their child, and is confident in their vaccination recommendations [20].

**Table 2:** Caregiver construct definitions.

Constructs and Definitions
Childhood vulnerability Risk of disease compared to the risk of potential adverse reactions [20].
Misleading information Information that has been manipulated to promote rumors related to vaccines [31].
Right to vaccination Caregivers have the ability to choose whether to vaccinate their child or to what degree their child is vaccinated [20].
Convenience Vaccine convenience is measured by the extent to which physical availability, affordability and willingness-to-pay, geographical accessibility, ability to understand (language and health literacy), and appeal of immunization services affect uptake [17].
Counseling satisfaction An interaction that results in a satisfied caregiver who feels informed and will return with their child to complete all vaccinations [20].
Trust The health provider is respectful of the caregiver’s opinions, is clear and understandable, cares about them and wants what is best for their child, and is confident in their vaccination recommendations [32].
Community responsibility The sense of responsibility the caregiver feels if the child becomes sick or infects others with a vaccine-preventable disease, and therefore their overall willingness to vaccinate to protect others [25,33].
Attitudes The extent to which people approve of vaccinations for their child, in what circumstances they find them acceptable [32].
Complacency Vaccine complacency exists where perceived risks of vaccine-preventable diseases are low, and vaccination is not deemed a necessary preventive action. Complacency about a particular vaccine or about vaccination, in general, is influenced by many factors, including other life/health responsibilities that may be seen to be more important at that point in time [17].
Confidence Trust in the effectiveness and safety of vaccines, the system that delivers them, including the reliability and competence of the health services and health professionals, and the motivations of the policymakers who decide on the needed vaccines [17].
Social norms Rules or expectations of vaccination status in a cultural or social group [32].
Decision-making A systematic process of choosing between vaccination or a set of alternatives based on specific criteria and the information available [32].
Sources of Information Persons, places, or things from where information about vaccinations comes from or where they are obtained [20].
Interpersonal communication (IPC) The exchange of information; in the case of counseling, this includes the transfer of biomedical or technical information and the complex roles and relationships within which health behaviors are negotiated [28].
Perceived threat Combined perceived seriousness and perceived susceptibility. Overall, it is a perception dangerous enough for the caregiver to take action [20].

## Data Availability

Not applicable.
